# Anti-Disturbance of Scale-Free Spiking Neural Network against Impulse Noise

**DOI:** 10.3390/brainsci13050837

**Published:** 2023-05-22

**Authors:** Lei Guo, Minxin Guo, Youxi Wu, Guizhi Xu

**Affiliations:** 1 State Key Laboratory of Reliability and Intelligence of Electrical Equipment, Hebei University of Technology, Tianjin 300130, China; guominxinxinxin@163.com (M.G.); gzxu@hebut.edu.cn (G.X.); 2 Hebei Key Laboratory of Bioelectromagnetics and Neuroengineering, School of Health Sciences and Biomedical Engineering, Hebei University of Technology, Tianjin 300130, China; 3 School of Artificial Intelligence, Hebei University of Technology, Tianjin 300401, China; wuc567@163.com

**Keywords:** brain-like model, spiking neural network, scale-free network, synaptic plasticity, anti-disturbance

## Abstract

The bio-brain presents robustness function to external stimulus through its self-adaptive regulation and neural information processing. Drawing from the advantages of the bio-brain to investigate the robustness function of a spiking neural network (SNN) is conducive to the advance of brain-like intelligence. However, the current brain-like model is insufficient in biological rationality. In addition, its evaluation method for anti-disturbance performance is inadequate. To explore the self-adaptive regulation performance of a brain-like model with more biological rationality under external noise, a scale-free spiking neural network(SFSNN) is constructed in this study. Then, the anti-disturbance ability of the SFSNN against impulse noise is investigated, and the anti-disturbance mechanism is further discussed. Our simulation results indicate that: (i) our SFSNN has anti-disturbance ability against impulse noise, and the high-clustering SFSNN outperforms the low-clustering SFSNN in terms of anti-disturbance performance. (ii) The neural information processing in the SFSNN under external noise is clarified, which is a dynamic chain effect of the neuron firing, the synaptic weight, and the topological characteristic. (iii) Our discussion hints that an intrinsic factor of the anti-disturbance ability is the synaptic plasticity, and the network topology is a factor that affects the anti-disturbance ability at the level of performance.

## 1. Introduction

The integration of brain science and brain-like intelligence will accelerate the development of information science and artificial intelligence [1]. Brain science provides the biological theoretical basis in the structure, function, and mechanism for brain-like intelligence [2]. The bio-brain presents a robustness function to external stimulus through its self-adaptive regulation and neural information processing [3]. Drawing from the advantages of the bio-brain to investigate the robustness function of a spiking neural network (SNN) is conducive to the advancement of brain-like intelligence. An SNN with neuron dynamics and synaptic weight dynamics has a strong ability to process nonlinear spatiotemporal information, which means the SNN is widely applied in the field of computational neuroscience [4,5,6]. The three basic elements of SNN construction are the neuron model, the synaptic plasticity model, and the network topology.

The neuron model with a dynamic process of spiking firing is the basic unit of information processing in an SNN. The Hodgkin–Huxley neuron model, as a fourth-order partial differential equation, can conform well to the neuro electrophysiological characteristics of bio-neurons [7]. However, its intrinsic computational complexity leads to high computing costs. In contrast, the Leaky Integrate-and-Fire neuron model, as a first-order partial differential equation, has low computational complexity but is inadequate when it comes to reflecting the neuroelectrophysiological characteristics of bio-neurons [8]. The Izhikevich neuron model, as a second-order partial differential equation, can reflect the neuroelectrophysiological characteristics of bio-neurons well. Additionally, due to the low computational complexity, the Izhikevich neuron model facilitates construction of large-scale networks [9]. Therefore, the Izhikevich neuron model is widely applied to construct SNNs [10,11,12,13].

The synaptic plasticity model is the regulatory rule of neural information transmission between neuron nodes in an SNN. Biological experiments have shown that excitatory synapses can strengthen the efficiency of neural information transmission [14,15]. The construction of SNNs based on the excitatory synaptic plasticity model has attracted the focus of scholars. For example, Mannan et al. constructed a neural circuit based on excitatory synaptic plasticity and demonstrated the biological effects through the efficient simulation of short-term facilitation and long-term potentiation [16]. The inhibitory synaptic plasticity can dynamically regulate information transmission in the aspects of speed, sensitivity, and stability, and forms the basis of information transmission together with excitatory synaptic plasticity [17,18]. A study by Pena et al. demonstrated that inhibitory synaptic plasticity is conducive to intermittent conversion between quiescent states and oscillatory states of neuron population in the SNN, which is similar to the asynchronous and synchronous cortical states [19]. Therefore, the synaptic plasticity model, in which excitatory synapses and inhibitory synapses co-regulate, has more biological rationality.

The network topology determines the connection forms between neuron nodes. Cognitive neuroscience demonstrates that bio-brain networks are complex networks with scale-free property [20,21]. According to complex network theory, the distribution of degree in a scale-free network conforms to power law distribution, which means the scale-free network has strong fault tolerance due to the non-uniformity of the degree distribution. With synthetic consideration of biological experimental results and topology characteristic analysis, computational neuroscientists have researched the SNNs with scale-free property. Research on the firing modes of bistable neurons coupled with electrical synapses and chemical synapses in a scale-free spiking neural network(SFSNN) has shown that the average firing rate of the neuron population presents non-monotonic behavior [22]. Zeraati et al. [23] investigated the self-organized criticality of an SFSNN regulated by the spike-timing-dependent plasticity (STDP) rule and found that dynamics of the SFSNN self-organized to the critical state of synchronization is achieved through the changes in degree distribution of the SFSNN. The brain-like networks with scale-free properties have biological rationality and theoretical basis. Furthermore, based on the complex network theory, the high-clustering networks have strong local information transmission ability due to the high aggregation degree. Combining the topological advantages of scale-free property and clustering characteristics, an SFSNN with a high-clustering characteristic can improve the information processing ability under the external stimulus.

Biological neuroscience has shown that the bio-brain has the ability to adaptively regulate external stimulus [24,25]. Drawing from the results of biological neuroscience, researchers have focused on the effect of the external stimulus on the firing characteristics of a brain-like network. Zhao et al. [26] investigated the firing characteristics of an SNN based on the Izhikevich neuron model under the external stimulus, and found that the white noise can induce stochastic resonance phenomena in the SNN. Etémé et al. [27] studied the firing activity and synchronization of an SNN stimulated by an external magnetic field, and found that electromagnetic induction can induce not only inerratic neuronal spiking firing, but also a synchronous firing mode of neurons. Research on energy coding under continuous stimulus in a fully connected SNN demonstrated that the energy distribution was positively and negatively correlated with the synaptic coupling strength and time delay in signal transmission, respectively [28]. Most studies have focused on the resonance, synchronization and neural coding of SNNs under external stimulus. Recently, researchers initiate to investigate the robustness function of the SNN under the external disturbance. For example, Guo et al. [29] studied the robustness function of the SNN under additive Gaussian white noise to classify digit images based on phase coding. Compared with the SNN without noise, the results show that the SNN under additive Gaussian white noise can maintain relatively high classification accuracy. This indicates that the SNN has the ability to suppress external disturbance. However, this kind of study is an indirect way to indicate the anti-disturbance of SNN. It is a challenge to establish a direct method to evaluate the anti-disturbance ability of a brain-like network and explore its anti-disturbance mechanism.

For synthetic consideration from the perspective of neuron, synapse and network topology, we construct a high-clustering SFSNN in this study. Then, we investigate the anti-disturbance ability of the high-clustering SFSNN against impulse noise. Finally, we discuss the anti-disturbance mechanism through the neural information dynamic evolution. The main contributions of our work are as follows.

(1) To improve the biological rationality of a brain-like model, a high-clustering SFSNN is constructed, which combines the topological advantages of scale-free property and a clustering characteristic.

(2) To evaluate the robustness function of the brain-like model, based on the two anti-disturbance indexes of the relative change rate of the firing rate and the correlation between membrane potential, we coherently verify that the SFSNN has an anti-disturbance ability against impulse noise, and the high-clustering SFSNN outperforms the low-clustering SFSNN in terms of anti-disturbance performance.

(3) Our discussion clarifies the neural information processing in the SFSNN under external noise and hints that an intrinsic factor of the anti-disturbance ability is the synaptic plasticity, and the network topology is a factor that affects the anti-disturbance ability at the level of performance.

This paper is organized as follows: A method to construct the SFSNN is proposed in Section 2. The anti-disturbance ability of the SFSNN is investigated and compared in Section 3. The anti-disturbance mechanism is discussed in Section 4. Finally, the conclusion is presented in Section 5.

## 2. Construction of SFSNN

A high-clustering SFSNN is constructed, in which the network topology is a scale-free network with a high clustering coefficient, the network node is presented by Izhikevich neuron model and the network edge is presented by the synaptic plasticity model regulated by excitatory and inhibitory synapses.

### 2.1. Generation of a Scale-Free Network

The Barrat Barthelemy Vespignani (BBV) algorithm models the dynamic growth of the local edge weights caused by adding new nodes in the generation process of a scale-free network [30]. The improved BBV algorithm has the characteristic of the original BBV algorithm. Meanwhile, it can adjust the clustering coefficients in a larger range [31]. Therefore, the improved BBV algorithm is employed to generate a scale-free network in this study. According to the improved BBV algorithm, the topology characteristics of the scale-free network can be changed by adjusting the connection probability *p* of new nodes, where p∈ (0,1]. The probability *p* rely on the total weight of the connected edges. The larger the total weight of connected edge, the higher the connection probability *p*.

We generate a scale-free network topology considering the scale-free property and the clustering coefficient by adjusting *p*.

(1) Scale-free property

The distribution of degree in a scale-free network conforms to a power law distribution, and the power law index γ is within the scope of [2, 3]. Node *k* connected to the other nodes conform to the following probability:

(1) $$P\left(k\right)∼k^{-\gamma }$$

(2) Clustering coefficient

The average clustering coefficient *C* can reflect the degree of network aggregation, which is defined as follows:

(2) $$C=\frac{1}{N}\sum_{i=1}^{N}\frac{2u_{i}}{e_{i}\left(e_{i}-1\right)}$$

where $u_{i}$ is the actual number of edges between nodes directly connected to node *i*, $e_{i}$ is the degree of node *i*, and *N* is the number of network nodes.

When *p* increases from 0.1 to 1.0 with the step length of 0.1, the power law index γ and the average clustering coefficients *C* are shown in Table 1.

**Figure 1:**
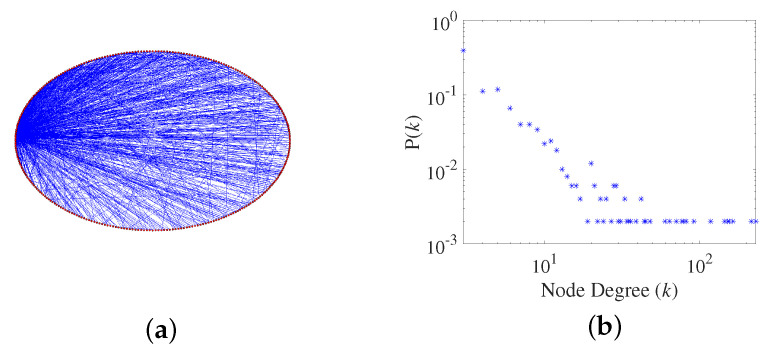
(**a**) Topology of the scale-free network with high clustering coefficient. The red points on the ellipse represent the network nodes. The blue lines inside the ellipse represent the network edges. (**b**) Distribution of degree. The x-coordinate is the degree of node, the y-coordinate is the probability of correspondence degree.

According to the definition of scale-free property, the γ should be within the scope of [2, 3]. Furthermore, bio-brain functional networks are complex networks with scale-free properties, and the γ of the human functional brain network is about 2 [32]. According to Table 1, when *p* is within the scope of [0.3, 0.9], the generated networks have scale-free properties. Therefore, *p* = 0.1, *p* = 0.2 and *p* = 1.0 are excluded, since their γ are out of the scope [2, 3]. Moreover, when *p* is 0.3, the γ of the network is 2.15, which conforms the most closely to the results of the biological experiment. In addition, when *p* is 0.3, the clustering coefficient of the network is 0.5001, which is the highest in the generated network with scale-free property. Therefore, *p* = 0.3 is chosen to generate a scale-free network with high clustering characteristics. Its topology and power law distribution are illustrated in Figure 1.

### 2.2. Izhikevich Neuron Model

**Figure 2:**
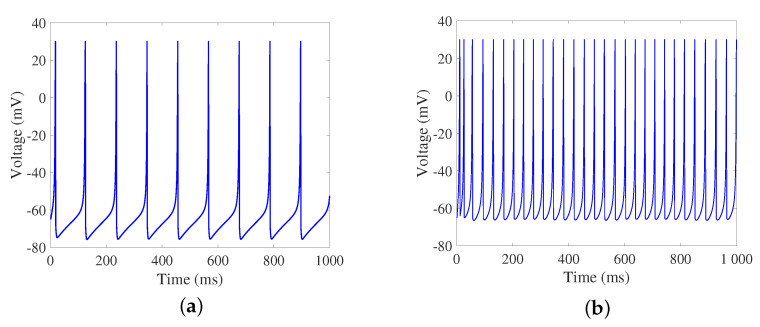
The firing modes of the Izhikevich neuron model. (**a**) Excitatory neurons. (**b**) Inhibitory neurons.

Due to the advantages of conforming to biological neuron firing characteristics and low computational complexity, the Izhikevich neuron model is introduced as network node of the SFSNN. The model is described as follows:

(3) $$\begin{array}{c} \left\{\begin{array}{c} \frac{dv}{dt}=0.04v^{2}+5v+140-u+I_{ext}+I_{syn}\\ \frac{du}{dt}=a\left(bv-u\right)\\ if\;v\geq 30,\;then\left\{\begin{array}{c} v\leftarrow c\\ u\leftarrow u+d \end{array}\right. \end{array}\right. \end{array}$$

where *v* is the membrane potential of neuron, *u* is the recuperative variate of *v*, $I_{ext}$ is the external input current, $I_{syn}$ is the sum of synaptic currents. *a*, *b*, *c*, and *d* are dimensionless parameters, and different firing modes of neurons can be obtained by adjusting their values. Regular spiking (RS) and low-threshold spiking (LTS) firing modes are taken as the excitatory and inhibitory neurons in this study. The parameter settings are as follows [9]: for excitatory neurons, *a* = 0.02, *b* = 0.2, *c* = −65, and *d* = 8; for inhibitory neurons, *a* = 0.02, *b* = 0.25, *c* = −65, and *d* = 2. The firing modes of the Izhikevich neuron model are illustrated in Figure 2.

### 2.3. Synaptic Plasticity Model

Combining excitatory synapses and inhibitory synapses, a synaptic plasticity model is introduced as the network edge. The model is described as follows:

(4) $$I_{syn}=g_{syn}\left(t\right)\left(E-V_{j}\left(t\right)\right)$$

where $I_{syn}$ s the synaptic current, $g_{syn}$ s the synaptic conductance, $V_{j}\left(t\right)$ is the membrane potential of a postsynaptic neuron and *E* is the reversal potential, including excitatory reversal potential $E_{ex}$ and inhibitory reversal potential $E_{in}$. Through synaptic conductance, the excitatory and inhibitory synaptic plasticity regulates the information transmission between presynaptic neurons and postsynaptic neurons. This regulation can be described as follows:

(1) When action potential from the presynaptic neuron *i* is not transmitted to the postsynaptic neuron *j*, the excitatory and inhibitory synaptic conductance decay exponentially, as follows:

(5) $$\tau_{ex}\frac{dg_{ex}}{dt}=-g_{ex},$$

(6) $$\tau_{in}\frac{dg_{in}}{dt}=-g_{in}$$

where $g_{ex}$ and $g_{in}$ are the excitatory synaptic conductance and inhibitory synaptic conductance, respectively; $\tau_{ex}$ and $\tau_{in}$ are the decay constants of $g_{ex}$ and $g_{in}$, respectively.

(2) When the action potential from the presynaptic neuron *i* is transmitted to the postsynaptic neuron *j*, then $g_{ex}$ and $g_{in}$ are regulated by the spike-timing-dependent plasticity rule, as follows:

(7) $$\left\{\begin{array}{c} g_{ex}\left(t\right)\to g_{ex}\left(t\right)+{\bar{g}}_{ex}\left(t\right)\\ {\bar{g}}_{ex}\to w_{ij}*g_{max} \end{array}\right.,w_{ij}=\left\{\begin{array}{c} A_{+}\;exp\left(\Delta t/\tau_{+}\right),\Delta t<0\\ -A_{-}\;exp\left(-\Delta t/\tau_{-}\right),\Delta t\geq 0 \end{array}\right.$$

(8) $$\left\{\begin{array}{c} g_{in}\left(t\right)\to g_{in}\left(t\right)+{\bar{g}}_{in}\left(t\right)\\ {\bar{g}}_{in}\to m_{ij}*g_{max} \end{array}\right.,m_{ij}=\left\{\begin{array}{c} -B_{+}\;exp\left(\Delta t/\tau_{+}\right),\Delta t<0\\ B_{-}\;exp\left(-\Delta t/\tau_{-}\right),\Delta t\geq 0 \end{array}\right.$$

where ${\bar{g}}_{ex}$ and ${\bar{g}}_{in}$ are the increments of $g_{ex}$ and $g_{in}$, respectively. $g_{max}$ is the upper limit on the synaptic weight. $w_{ij}$ and $m_{ij}$ are the excitatory synaptic correction functions and inhibitory synaptic correction functions, respectively. $A_{+}$ and $A_{-}$ are the maximum correction values and minimum correction values of the $g_{ex}$, respectively. $B_{+}$ and $B_{-}$ are the maximum correction values and minimum correction values of the $g_{in}$, respectively. $\tau_{+}$ and $\tau_{-}$ represent the neuronal firing interval when the $g_{syn}$ is strengthened and weakened, respectively. Δt is the neuronal firing interval between the firing moment of presynaptic neuron and postsynaptic neuron.

In this study, the parameter settings are as follows [33]: $E_{ex}$ = 0 mV, $E_{in}$ = −70 mV, $\tau_{ex}$ = $\tau_{in}$ = 5 ms, $g_{max}$ = 0.015, $A_{+}$ = 0.1, $A_{-}$ = 0.105, $B_{+}$ = 0.02, $B_{-}$ = 0.03, and $\tau_{+}$ = $\tau_{-}$ = 20 ms.

### 2.4. Construction Process of the SFSNN

Our simulation is carried out on a PC with a 4.9 GHz Intel(R) Core i7-9700k CPU and 16 GB RAM. We conducted simulations to observe the anti-disturbance ability of the SFSNNs with different network sizes of 500, 800 and 1000, and found that there was no obvious difference among them. In addition, based on the results of mammalian cortical neuroanatomy [34], we randomly selected 80% of the neurons and set the parameters to excitatory neurons, while for the remaining 20%, we set the parameters to inhibitory neurons. The construction process of an SFSNN according to Algorithm 1 is as follows.
**Algorithm 1** The construction algorithm of the SFSNN**Input:** Adjacency matrix of the scale-free network at P=0.3
**Output:** The high-clustering SFSNN
1:Add the Izhikevich neuron model (Equation (3));2:n←500    //size of our SFSNN is 500 nodes3:**for** i=1 to *n* **do**4:    A←rand(n,1);5:    **if** A(i)<=0.8 **then**    //select 80% of Izhikevich neuron models6:        Set parameters of *a*, *b*, *c*, and *d* to excitatory neurons (Equation (3));7:    **else**   //for other 20% of Izhikevich neuron models8:        Set parameters of *a*, *b*, *c*, and *d* to inhibitory neurons (Equation (3));9:    **end if**10:**end for**11:Add the synaptic plasticity model (Equation (4));    // connect the neuron models12:**if** neuron *j* at the front of the synapse does not receive an action potential from neuron *i* behind the synapse **then**13:    Calculate *g* using Equations (5) and (6);14:**else**15:    Calculate *g* using Equations (7) and (8);16:**end if**17:**if** $g<g_{min}$ **then**18:     $g\leftarrow g_{min}$;19:**else**20:     $g\leftarrow g_{max}$;21:**end if**22:**return** SFSNN;


## 3. Anti-Disturbance of the SFSNN

The anti-disturbance ability of the high-clustering SFSNN against impulse noise is investigated based on two indexes of the relative change rate of firing rate and the correlation between the membrane potential. Furthermore, the anti-disturbance performance of the high-clustering SFSNN and the low-clustering SFSNN is compared under impulse noise.

### 3.1. External Disturbance and Anti-Disturbance Indexes

1. Impulse noise
  Impulse noise is an irregular discontinuous signal composed of pulse spikes, which is characterized by short duration, large amplitude and burst. It can be described as follows:
  (9) $$s\left(t\right)=\left\{\begin{array}{c} A_{s},t\in \left[T_{0},T_{0}+T\right]\\ 0,else \end{array}\right.$$
  where $A_{s}$ is the intensity of impulse noise, $T_{0}$ is the start time of the stimulus and *T* is the duration of the stimulus. In this study, impulse noise s(t), as current disturbance, is applied to $I_{e}xt$ in Equation (3) of all neurons in the SFSNN.
2. The indexes of anti-disturbance
  (1) The relative change rate of the firing rate
    The firing rate of a neuron reflects the frequency of action potentials per unit of time in a neuron. The relative change rate of the firing rate δ can characterize the change degree of the neuronal firing rate before and after disturbance, which is defined as follows:
    (10) $$\delta =\frac{\left|f_{j}-f_{i}\right|}{f_{i}}*100\%$$
    where $f_{i}$ and $f_{j}$ are the mean firing rates of the SFSNN before and after disturbance, respectively. The smaller the value of δ, the smaller the changes in the neuronal firing rate before and after disturbance, and the better the anti-disturbance ability of the SFSNN.
  (2) The correlation between membrane potential
    The correlation between membrane potential ρ reflects the degree of similarity between the membrane potentials of the neurons before and after disturbance, which is defined as follows:
    (11) $$\rho \left(\tau \right)=\frac{\sum_{t=t_{1}}^{t_{2}-\tau +1}x_{i}\left(t\right)x_{j}\left(t+\tau \right)}{\sqrt{\sum_{t=t_{1}}^{t_{2}-\tau +1}x_{i}^{2}\left(t\right)\sum_{t=t_{1}}^{t_{2}-\tau +1}x_{j}^{2}\left(t+\tau \right)}}$$
    where $x_{i}$ and $x_{j}$ are the mean membrane potential of neurons in the SFSNN before and after disturbance, respectively, and $\left[t_{1},\;{\;t}_{2}\right]$ is the simulation time. The larger the value of ρ, the smaller the changes in the neuronal membrane potential before and after disturbance, and the better the anti-disturbance ability of the SFSNN.

### 3.2. Anti-Disturbance Ability of the SFSNN

**Figure 3:**
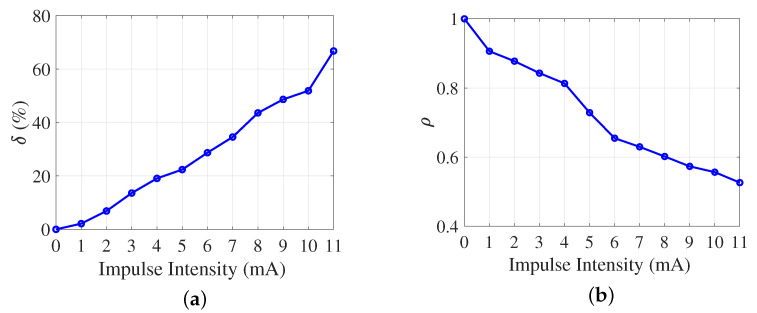
Anti-disturbance ability of the SFSNN against impulse noise. (**a**) δ. (**b**) ρ.

To investigate the anti-disturbance ability, impulse noise is applied to all neurons in the SFSNN. The intensity scope of impulse noise is [0, 11] mA with steps of 1 mA. The anti-disturbance ability of the SFSNN against impulse noise is evaluated from the two perspectives of δ and ρ. The results are illustrated in Figure 3.

According to Figure 3a, δ of the SFSNN presents an upward trend with the increase in impulse intensity. When impulse intensity is within [0, 4] mA, δ does not exceed 20%. When impulse intensity is within [5, 11] mA, δ increases from 22.36% to 66.73%. According to Figure 3b, with the increase in impulse intensity, ρ of the SFSNN presents a downward trend. When the impulse intensity is within [0, 4] mA, ρ is larger than 0.8. When the impulse intensity is within [5, 11] mA, ρ decreases gradually from 0.7286 to 0.5261. Combining δ and ρ, the results demonstrate that the high-clustering SFSNN has an anti-disturbance ability against impulse noise, and the anti-disturbance ability decreases gradually as the impulse intensity increases.

### 3.3. Comparison of Anti-Disturbance Ability

**Figure 4:**
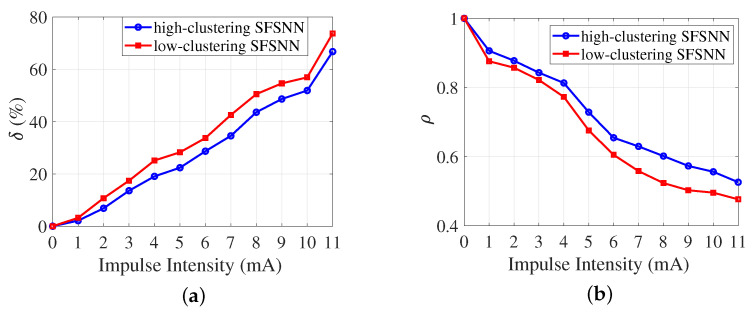
Comparison of the anti-disturbance ability of the two SFSNNs against impulse noise. (**a**) δ. (**b**) ρ.

To comparatively analyze the effect of different clustering coefficients on anti-disturbance ability, we construct an SFSNN with a low clustering coefficient (low-clustering SFSNN) by employing the Barabási Albert (BA) algorithm [35], which has the same scale-free property as the high-clustering SFSNN above. According to Equation (2), the average clustering coefficient of the scale-free network based on the BA algorithm is 0.0921. The anti-disturbance ability against impulse noise is evaluated by δ and ρ. The comparison results of the anti-disturbance ability of the two SFSNNs are illustrated in Figure 4.

According to Figure 4a, δ of the two SFSNNs represent similar trends; that is, δ increases gradually with the increase in the impulse intensity. In addition, δ of the high-clustering SFSNN is always lower than δ of the low-clustering SFSNN. According to Figure 4b, ρ of the two SFSNNs represent similar trends; that is, ρ decreases gradually with the increase in the impulse intensity. In addition, ρ of the high-clustering SFSNN is always higher than ρ of the low-clustering SFSNN.

To further verify the significant statistical difference between the anti-disturbance ability of the two SNNs, the Wilcoxon test is employed to conduct a significance test on δ and ρ of two SNNs under impulse noise. The test steps are as follows.

(1) Calculate the difference *w* between two samples. If *w* is a positive number, denote it as a positive sign; otherwise, *w* is a negative number, denoted it as a negative sign.
(2) Calculate the corresponding order by sorting the absolute value of *w*.
(3) Calculate the sum order of the positive and negative signs *w*, denoted as $w^{+}$ and $w^{-}$, respectively.

In this study, the significance levels of δ and ρ for two SNNs are 0.0028 and 0.0033, respectively. Both significance levels are below 0.05. These results indicate that the significant statistical difference exist in the anti-disturbance ability of the two SNNs under impulse noise.

In summary, from two perspectives of δ and ρ, our simulation results coherently verify that the anti-disturbance ability of the high-clustering SFSNN outperforms that of the low-clustering SFSNN.

## 4. Discussion

To explore the anti-disturbance mechanism of the SFSNN, the neural information processing in SFSNNs under impulse noise is discussed, which involves the evolution process of the neuronal firing rate, the synaptic weight, and the topological characteristics.

### 4.1. Firing Rate

**Figure 5:**
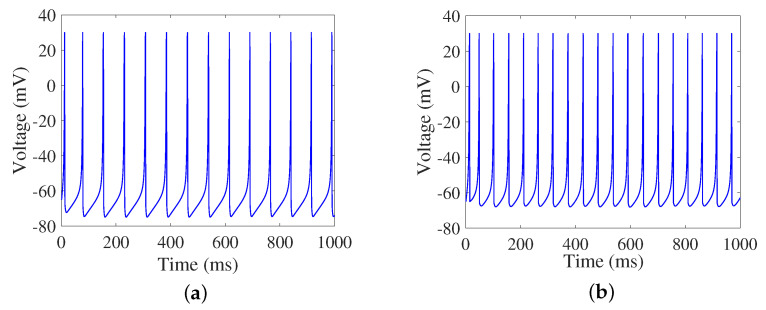
The firing modes of Izhikevich neuron under impulse noise. (**a**) excitatory neurons. (**b**) inhibitory neurons.

Impulse noise with 4 mA as an example is applied to all neurons in the SFSNN. The firing modes of a single Izhikevich neuron after disturbance are illustrated in Figure 5.

**Figure 6:**
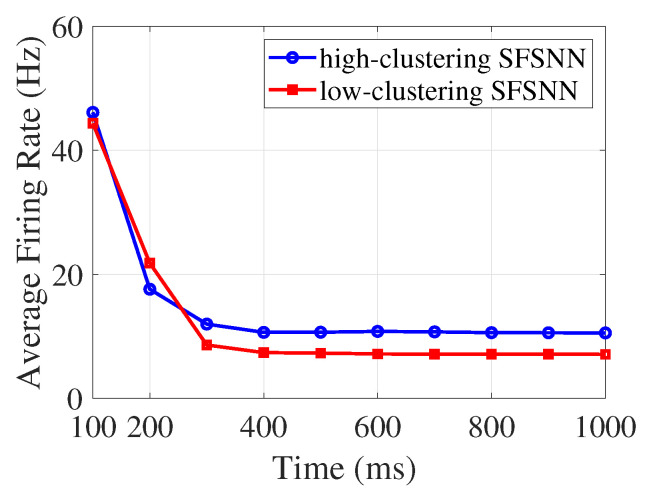
The evolution process of the average firing rate under impulse noise.

Compared with Figure 2, we find that the firing rate changes obviously under external noise, which indicates that external noise can affect the firing activity of neurons. The average firing rate of the SFSNN at a given moment is characterized by the mean of firing rate of all neurons in the network during a 100 ms time window. The evolution process of the average firing rate in the high-clustering SFSNN and the low-clustering SFSNN under impulse noise is illustrated in Figure 6.

According to Figure 6, the evolution process of the average firing rate in high-clustering SFSNN and low-clustering SFSNN under impulse noise is different. However, the two SFSNNs show similar evolutionary trends: during the first 300 ms, the average firing rate of SFSNNs with different clustering coefficients decreases sharply; after 300 ms, the average firing rate decreases tardily and gradually stabilizes.

### 4.2. Synaptic Weight

**Figure 7:**
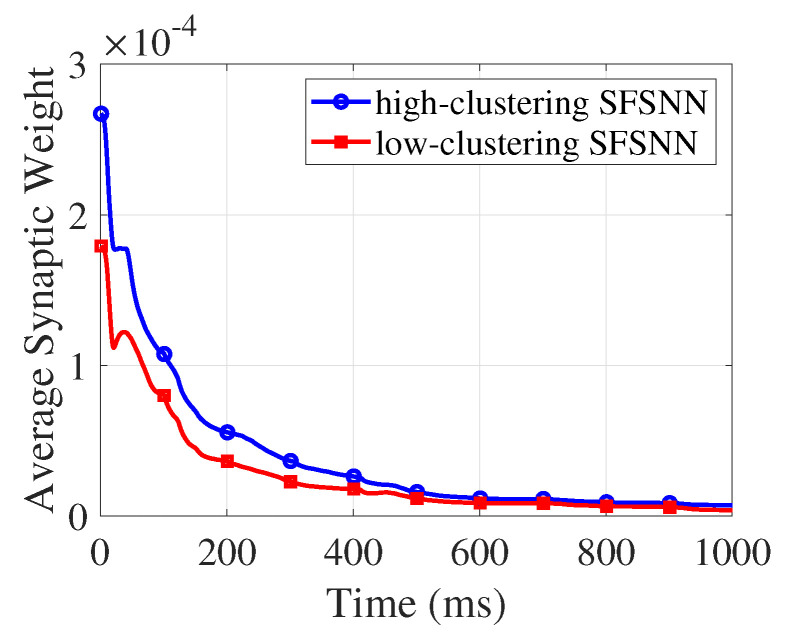
The evolution process of the average synaptic weight under impulse noise.

According to Equations (6) and (7), the changes in synaptic weight depend on the firing moment of the presynaptic neurons and postsynaptic neurons. Therefore, changes in the neuronal firing rate can induce changes in synaptic weight. The average synaptic weight is the mean of all the synaptic weights in the SFSNNs. The evolution process of the average synaptic weight in the SFSNNs under impulse noise is illustrated in Figure 7.

According to Figure 7, the evolution process of the average synaptic weight in high-clustering SFSNN and low-clustering SFSNN under impulse noise is different. However, the two SFSNNs show similar evolutionary trends: during the first 400 ms, the average synaptic weight of SFSNNs with different clustering coefficients decreases sharply; after 400 ms, the average synaptic weight decreases tardily and gradually stabilizes.

### 4.3. Relevance between the Synaptic Plasticity and the Anti-Disturbance Ability

To explore the anti-disturbance mechanism of the SFSNNs, we conduct an association analysis to establish the relevance between the synaptic plasticity and the anti-disturbance ability based on the Pearson correlation coefficient.

#### 4.3.1. Pearson Correlation Coefficient and *t*-Test

The Pearson correlation coefficient can characterize a statistical correlation between two samples *X* and *Y*. The correlation coefficient *R* is defined as follows:

(12) $$R=\frac{\sum_{i=1}^{n}\left(X_{i}-\bar{X}\right)\left(Y_{i}-\bar{Y}\right)}{\sqrt{\sum_{i=1}^{n}{\left(X_{i}-\bar{X}\right)}^{2}}\sqrt{\sum_{i=1}^{n}{\left(Y_{i}-\bar{Y}\right)}^{2}}}$$

The more adjacent the |R| is to 1, the more significant the correlation is. By contrast, the more adjacent the |R| is to 0, the less significant the correlation is. A $t_{test}$ is carried out to investigate the significance of a sample *R* to the totality, which is defined as follows:

(13) $$t_{test}=\frac{R}{\sqrt{\left(1-R^{2}\right)/\left(n-2\right)}}$$

For the *t*-test, a significance level of 0.01 is indicated by “**”, and a significance level of 0.05 is indicated by “*”.

#### 4.3.2. Evolution Process of the Anti-Disturbance Ability

**Figure 8:**
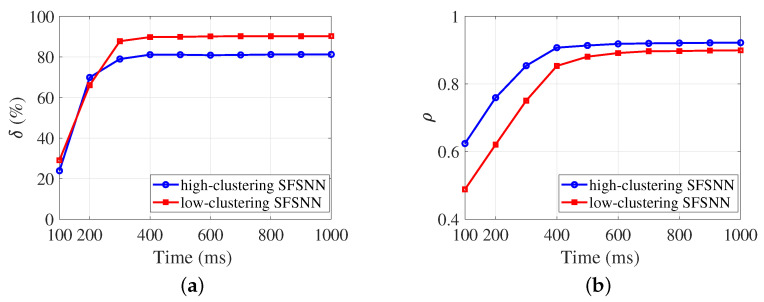
The evolution process of the anti-disturbance ability under impulse noise. (**a**) δ. (**b**) ρ.

To explore the reason for the anti-disturbance ability, the evolution process of the anti-disturbance ability of the SFSNNs under impulse noise is illustrated in Figure 8.

According to Figure 8, δ and ρ of the high-clustering SFSNN and the low-clustering SFSNN show similar evolutionary trends, respectively. In Figure 8a, at the initial 300 ms, δ changes sharply and then gradually stabilizes. In Figure 8b, at the initial 400 ms, ρ changes sharply and then gradually stabilizes.

#### 4.3.3. Relevance Analysis

In this study, according to Equation (12), *X* is δ or ρ, *Y* is the average synaptic weight. The correlation analysis between the anti-disturbance indexes and the average synaptic weight of the high-clustering SFSNN and the low-clustering SFSNN are shown in Table 2.

According to Table 2, the average synaptic weight and the values of δ and ρ are significantly correlated at a 0.01 significant level (two-sided *t*-test) for both the high-clustering SFSNN and the low-clustering SFSNN. The correlation results demonstrate that the anti-disturbance ability is intimately related to the dynamic regulation of synaptic plasticity. Simulation results hint that an intrinsic factor of the anti-disturbance ability is the synaptic plasticity.

### 4.4. Effect of Network Topology on the Anti-Disturbance Ability

The clustering coefficient can reflect the aggregation degree and affect the information transmission of a network. We analyze the impact of topology on anti-disturbance ability using weighted clustering coefficient.

#### 4.4.1. Weighted Clustering Coefficient

The weighted clustering coefficient ${\tilde{C}}_{i}$ in a network is defined as follows [30]:

(14) $$\begin{array}{c} {\tilde{C}}_{i}=\frac{1}{s_{i}\left(k_{i}-1\right)}\sum_{j,k}\frac{\left(g_{ij}+g_{ik}\right)}{2}a_{ij}a_{jk}a_{ki} \end{array}$$

where $g_{ij}$ and $g_{ik}$ are the synaptic weights, $k_{i}$ is the degree of node *i* and $s_{i}$ is the strength of node *i*, $a_{ij}$ is the adjacency matrix.

#### 4.4.2. Evolution Process of the Weighted Clustering Coefficient

**Figure 9:**
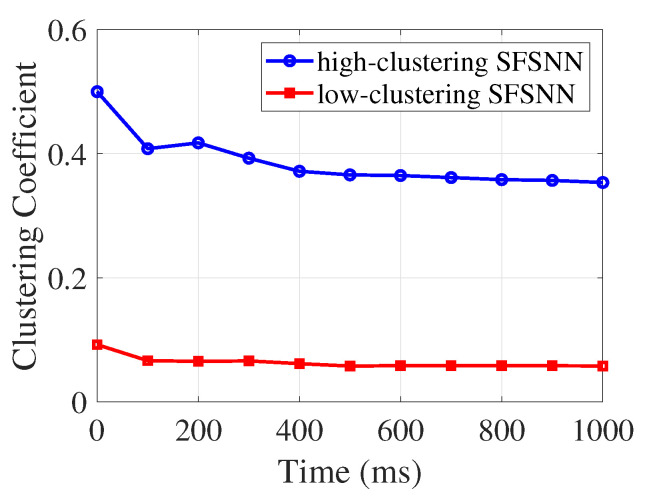
The evolution process of the clustering coefficient under impulse noise.

In this study, according to Equation (14), the changes in synaptic weight can induce changes in the clustering coefficient of the SFSNNs. The evolution process of the clustering coefficient in the high-clustering SFSNN and the low-clustering SFSNN under impulse noise is illustrated in Figure 9.

According to Figure 9, the clustering coefficient of the high-clustering SFSNN invariably remains much higher than that of the low-clustering SFSNN under impulse noise. During the stimulus, the clustering coefficients of the two SFSNNs decrease slightly, and then gradually stabilize. It can be seen from the construction process that the node and edge models of the two SFSNNs are the same. In terms of topology, the topologies of the two SFSNNs have the same power law index, but quite different clustering coefficients. This difference leads to the different anti-disturbance performances of the two SFSNNs. The simulation results hint that the network topology affects the anti-disturbance ability at the level of performance.

Combining the discussion above, the neural information processing in the SFSNN under external noise is clarified, which is a dynamic chain effect of the neuron firing, the synaptic weight, and the topological characteristic. The specific process is as follows: impulse noise affects the firing activity of neurons, leading to changes in the firing rate, which induces changes in the synaptic weights according to Equations (4)–(8). Then, changes in the synaptic weights lead to changes in the clustering coefficient according to Equation (14), which further affects the anti-disturbance ability at the level of performance. The chain effect of the neuron firing, the synaptic weight, and the topological characteristic form neural information processing in the SFSNN under external noise.

## 5. Conclusions

To explore the self-adaptive regulation performance of a brain-like model with more biological rationality under external noise, a high-clustering SFSNN is constructed in this study, in which the network node is presented by the Izhikevich neuron model, the network edge is presented by a synaptic plasticity model regulated by excitatory synapses and inhibitory synapses and the network topology is a scale-free network with a high clustering coefficient. The anti-disturbance ability of the SFSNN is evaluated based on two anti-disturbance indexes. The anti-disturbance mechanism is discussed through the neural information dynamic evolution. The simulation results show that: (i) From the two perspectives of δ and ρ, we coherently verify that the SFSNN has anti-disturbance ability against impulse noise and the high-clustering SFSNN outperforms the low-clustering SFSNN in terms of anti-disturbance performance. (ii) The neural information processing in the SFSNN under external noise is clarified, which is a dynamic chain effect of the neuron firing, the synaptic weight and the topological characteristic. Impulse noise induces changes in neuronal firing activity, which leads to the dynamic regulation of synaptic plasticity, and then changes the network topology. (iii) Correlation analysis shows that the anti-disturbance indexes are intimately related to the dynamic regulation of synaptic weight, which hints that an intrinsic factor of the anti-disturbance ability is synaptic plasticity. Moreover, topological analysis shows that network topology is a factor that affects the anti-disturbance ability at the level of performance. 

## Figures and Tables

**Table 1:** Topological property of the scale-free network with different *p*.

*p*	0.1	0.2	0.3	0.4	0.5	0.6	0.7	0.8	0.9	1.0
γ	1.55	1.82	2.15	2.41	2.51	2.76	2.86	2.87	2.98	3.18
*C*	0.7028	0.6236	0.5001	0.4707	0.4180	0.3884	0.3133	0.2524	0.1889	0.1643

**Table 2:** Pearson correlation coefficients between the average synaptic weight and anti-disturbance indexes.

Types of SFSNN	High-Clustering	Low-Clustering
δ	−0.915 **	−0.988 **
ρ	−0.970 **	−0.963 **

## Data Availability

Not applicable.
